# E-Cadherin Is Transcriptionally Activated via Suppression of ZEB1 Transcriptional Repressor by Small RNA-Mediated Gene Silencing

**DOI:** 10.1371/journal.pone.0028688

**Published:** 2011-12-21

**Authors:** Minami Mazda, Kenji Nishi, Yuki Naito, Kumiko Ui-Tei

**Affiliations:** Department of Biophysics and Biochemistry, Graduate School of Science, University of Tokyo, Tokyo, Japan; Keio University, Japan

## Abstract

RNA activation has been reported to be induced by small interfering RNAs (siRNAs) that act on the promoters of several genes containing E-cadherin. In this study, we present an alternative mechanism of E-cadherin activation in human PC-3 cells by siRNAs previously reported to possess perfect-complementary sequences to E-cadherin promoter. We found that activation of E-cadherin can be also induced via suppression of ZEB1, which is a transcriptional repressor of E-cadherin, by seed-dependent silencing mechanism of these siRNAs. The functional seed-complementary sites of the siRNAs were found in the coding region in addition to the 3′ untranslated region of ZEB1 mRNA. Promoter analyses indicated that E-boxes, which are ZEB1-binding sites, in the upstream promoter region are indispensable for E-cadherin transcription by the siRNAs. Thus, the results caution against ignoring siRNA seed-dependent silencing effects in genome-wide transcriptional regulation. In addition, members of miR-302/372/373/520 family, which have the same seed sequences with one of the siRNAs containing perfect-complementarity to E-cadherin promoter, are also found to activate E-cadherin transcription. Thus, E-cadherin could be upregulated by the suppression of ZEB1 transcriptional repressor by miRNAs in vivo.

## Introduction

Small RNA molecules, including small interfering RNAs (siRNAs) and microRNAs (miRNAs), are crucial regulators of posttranscriptional gene silencing (PTGS) referred to as RNA silencing or RNA interference (RNAi). RNAi is an evolutionarily conserved pathway induced by siRNAs, 21–23-nucleotide (nt) double-stranded RNAs (dsRNAs) with 2-nt 3′ overhangs. The siRNAs incorporated into cells are transferred to an RNAi effector complex called the RNA-induced silencing complex (RISC) [1], [2]. The RISC assembles on one of the two strands of the siRNA duplex and is activated upon the removal of the passenger strand [3]–[5]. The activated RISC is a ribonucleoprotein complex minimally consisting of the core protein Argonaute (Ago) and single-stranded siRNA, which acts as a guide to recognize mRNAs with sequence complementarity [6]–[8]. Usually, a guide strand of siRNA forms base-pairing with perfectly complementary mRNA sequences in siRNA-mediated RNAi. However, it can also give rise to the silencing of other genes with incompletely complementary sequences. This phenomenon is referred to as the seed-dependent off-target effect. A growing body of evidence from genome-wide experiments indicates that the target recognition mechanism is similar to that of miRNA-mediated gene silencing. The transcripts with sequences complementary to the seed region, nucleotide positions 2–8 from the 5′ end of the RISC-loaded siRNA strand or miRNA, are mainly reduced [9]–[14]. This is probably because the seed nucleotides are present on the surface of Ago in a quasi-helical form to serve as the entry or nucleation site for small RNAs in the RISCs [14]–[16].

Small RNAs are also known to induce transcriptional gene silencing (TGS). Originally, TGS was reported in plants and yeast [17], [18], in which small RNAs induce silencing of specific genes containing homologous sequences at the transcriptional level. Recently, TGS was shown to be induced in human cells by exogenous siRNAs that have sequence complementarity to the genomic DNA in the promoter regions of several genes containing Elongation factor-1 (EF1A) in 293FT cells [19]–[21], E-cadherin (CDH1) in HCT116 cells [22], immunodeficiency virus-1 (HIV-1) coreceptor (CCR5) in HEK293T cells [23], progesterone receptor (PR) in T47D cells [24], [25], ubiquitin C (UbC) in 293Gt cells [26], and vascular endothelial growth factor (VEGF) in HeLa and ARPE-19 cells [27]. Recent studies demonstrated that TGS is associated with biochemical modification of critical residues in the histone tails and silent-state chromatin marks, not only at the target promoter region but also at the downstream region, which in turn is associated with repressive chromatin structures [20], [23], [28], [29]. The protein components responsible for TGS were partially identified and found to contain Ago proteins, DNA methyltransferase 3a (DNMT3a), DNMT1, and histone deacetylase (HDAC1) [20], [21], [23], [25], [26], [29]. Although DNA methylation in siRNA-directed TGS has not been observed in mammalian cells [22], the process is considered to operate through epigenetic modifications similar to those in plants and yeast.

Confusingly, transcriptional activation was shown to be directed by siRNAs with promoter complementarity in a process referred to as RNA activation (RNAa), which is reported to be induced by siRNAs with perfect complementarity to the promoter sequences of genes containing E-cadherin or p21^WAF/CIP1^ in PC-3 and MCF-7 cells, VEGF in HeLa cells [30], and PR in T47D and MCF7 cells [31], [32]. Although the precise mechanism of RNAa remains uncertain, the process requires the Ago protein and is associated with the 5′ region of the siRNA guide strand overlapping seed region [31], [32]. These are analogous to the already known mechanism of RNA silencing. Thus, a possible alternative pathway of RNAa is considered that siRNA seed-dependent silencing on a transcriptional repressor causes the activation of downstream genes. Recently, siRNA targeted to the HIV-1 LTR promoter was demonstrated to induce gene activation via an indiscriminate off-target effect by suppressing the C10orf76 transcriptional regulator candidate [33].

In this study, we investigated whether the seed-dependent off-target effects of siRNA also induce transcriptional activation in a human endogenous gene. E-cadherin is a central component of cell–cell adhesion junctions required for the formation of epithelia, and shown to be linked to the control of embryonic stem cell (ESC)-like pluripotency [34], [35]. Since the transcriptional regulation of E-cadherin has been well studied, we examined the seed-dependent off-target silencing effects of the RNAa-inducible and E-cadherin promoter-directed siRNAs, dsEcad215, dsEcad320, and dsEcad640 [30], and miR-373 [36], which has the same seed sequence with dsEcad640, reported previously [30], [36]. Our results showed that one of the alternative mechanisms for activating E-cadherin expression involves the seed-dependent off-target effects of these siRNAs on the Zinc finger E-box binding homeobox 1 (ZEB1) transcriptional repressor of E-cadherin. When ZEB1 was downregulated by seed-dependent siRNA silencing, E-cadherin transcription was upregulated. Furthermore, the members of miR-302a/372/373/520 family miRNAs, which contain the same seed sequence with dsEcad640, showed parallel effects on ZEB1 expression, advocating the possibility that such a mechanism is also functional *in vivo*.

## Results

### Seed-dependent off-target effects of siRNAs with perfect complementarity to the E-cadherin promoter

**Figure 1:**
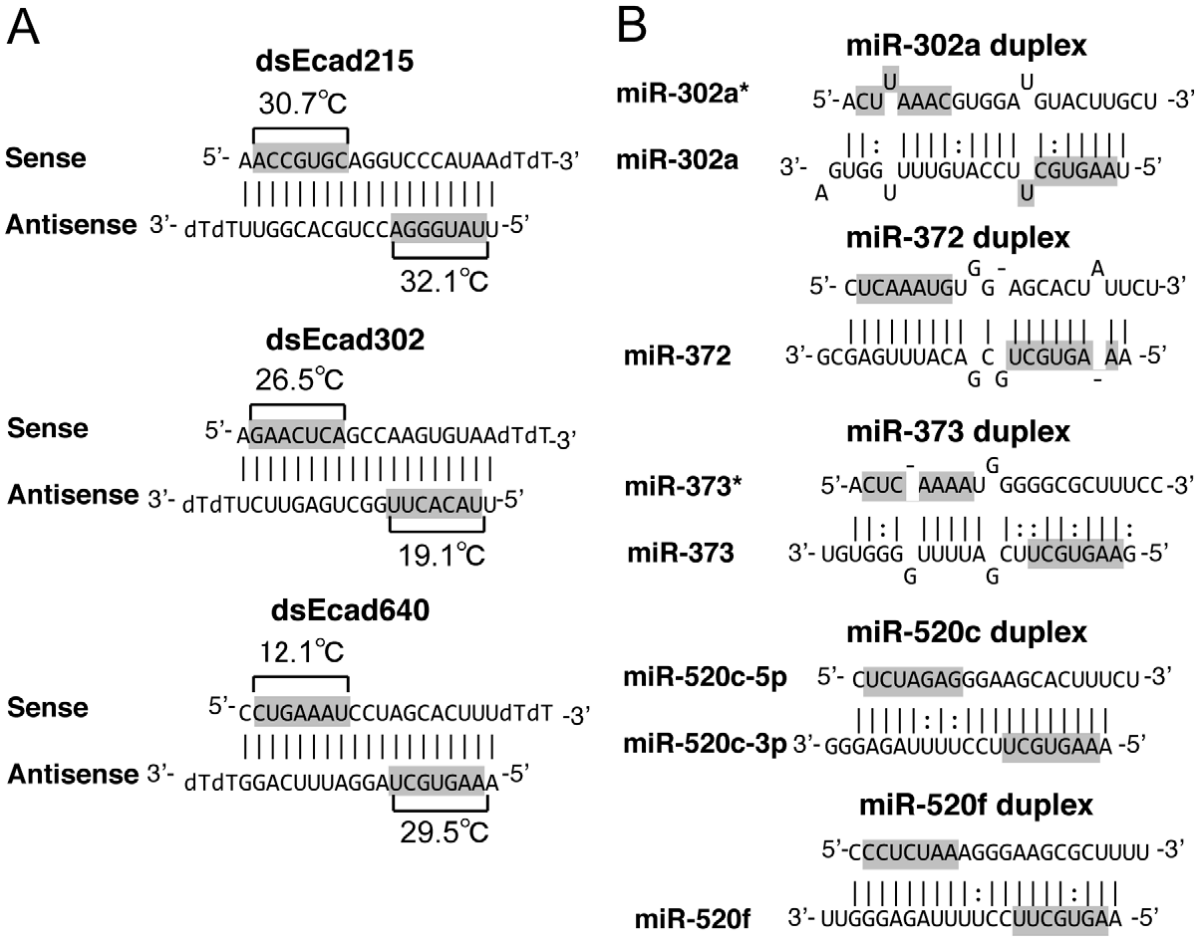
Structures and sequences of siRNAs and miRNAs. Structures and sequences of siRNAs (A) and miRNAs (B) used in this study. The same siRNAs shown in Li et al [30], [36], dsEcad215, dsEcad302, and dsEcad640, were chemically synthesized. Their guide strands are shown at the lower sides, and the passenger strands at the upper sides. The miRNAs were synthesized to form same structures shown in miRBase [51]. The miR-302a duplex is composed of miR-302a and miR-302*; the miR-373 duplex, miR-373 and miR-373*; and the miR-520c duplex, miR-520c-5p and miR-520c-3p. The opposite strand miRNAs of miR-372 and miR-520f are not annotated. The seed region is marked in gray. The T_m_ values of siRNA seed duplexes are shown in (A). The solid lines indicate Watson–Crick base pairing; dotted lines indicate G∶U wobble pairing.

**Figure 2:**
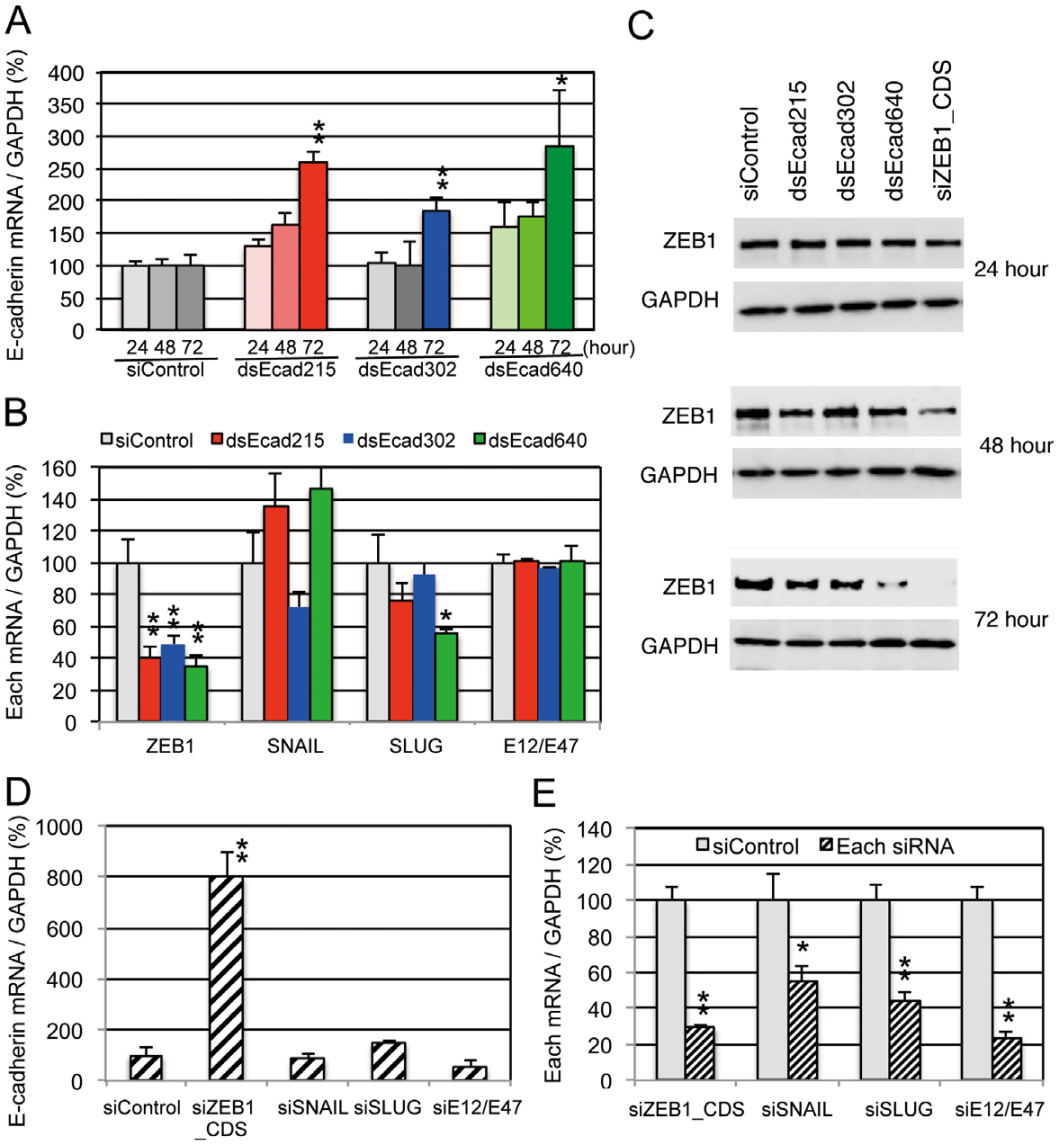
The effects of dsEcad215, dsEcad302, and dsEcad640 on E-cadherin and its transcriptional repressors in PC-3 cells. (A) Effects of dsEcad215, dsEcad302, and dsEcad640 (5 nM) on E-cadherin expression at 24, 48, and 72 hour after transfection. (B) Effects of dsEcads on the expression of E-cadherin transcriptional repressors, ZEB1, SNAIL, SLUG, and E12/E47 at 72 hour after transfection. (C) Western blot analyses of the ZEB1 protein expression at 24, 48, and 72 hour after transfection. (D) Effect of knockdown of each transcriptional repressor on E-cadherin activation at 72 hour after transfection. (E) Knockdown efficiency of each siRNA. P-values were determined by Student's t-test (▪; P<0.01, *; P<0.05).

In human PC-3 cells, the siRNAs named as dsEcad215, dsEcad302, and dsEcad640 (Figure 1A), which contain sequences with perfect complementarity to the promoter region of the E-cadherin gene, were reported to induce transcriptional activation of E-cadherin [30]. Upregulation of E-cadherin expression was not significant at 24 and 48 hour after transfection, but it was significantly upregulated certainly confirmed by dsEcad215, dsEcad302, and dsEcad640 at 72 hour after transfection (Figure 2A). However, the alternative possibility was considered that seed-dependent off-target effect of siRNA could activate E-cadherin transcription via suppression of its negative transcription factor(s). The seed-dependent off-target effect is shown to be induced by complementarity in the 7-nt seed region mainly positioned 2–8 from the 5′ end of the siRNA guide strand [14], [37], [38]. Among the known negative regulators of E-cadherin, ZEB1 [39], ZEB2 [40], SNAIL [41], SLUG [42], and E12/E47 [43] were found to have one or more complementary sequence(s) of seed regions positioned 2–8 or 1–7 of sense or antisense strand of dsEcad215, dsEcad302, or dsEcad640 in their mRNAs (Table S1). At 72 hour after transfection of each of the chemically synthesized dsEcads into PC-3 cells, mRNA levels of these repressors were measured by quantitative RT-PCR (qRT-PCR). ZEB1 mRNA has at least two seed-complementary sequences of each dsEcad (Table S1). Accordantly, the expression level of ZEB1 mRNA was significantly reduced to 35–48% by the transfection of dsEcad215, dsEcad302, and dsEcad640 (Figure 2B). The expression level of SLUG mRNA was also significantly reduced by the transfection of dsEcad640, but the seed-complementary site to dsEcad640 was not found (Table S1), suggesting that the reduction of SLUG expression is not induced by the direct effect of dsEcad640 but the secondary effect probably caused by the inhibition of the other upstream regulator(s). In contrast, although seed-complementary site of either dsEcad215 or dsEcad302 was found in SLUG mRNA, dsEcad215 and dsEcad302 showed weak but not significant silencing activities. No significant changes in the expression levels of SNAIL or E12/E47 were observed by any dsEcads, although both of them have a seed-complementary site to dsEcad640 sense strand. These results suggested that all of dsEcad215, dsEcad302, and dsEcad640 could downregulate the expression level of a transcriptional repressor, ZEB1, by seed-dependent silencing effects accidentally, but could not suppress the other repressors at least in PC-3 cells. The changes of ZEB1 protein levels were also examined by Western blot (Figure 2C). At 24 hour after transfection, no or little downregulation of ZEB1 protein was observed by dsEcads and siRNA targeting to ZEB1 coding region (siZEB1_CDS). The noticeable level of decrease was observed by siZEB1_CDS at 48 hour, and the slight decrease was detected by dsEcad215 or dsEcad640. At 72 hour, ZEB1 protein was severely repressed by siZEB1_CDS, and the unambiguous repression by dsEcad215, dsEcad302, or dsEcad640 was also observed. To investigate the involvement of each repressor in E-cadherin activation, knockdown experiments using siRNAs targeting each of the transcriptional repressors were performed. The expression level of E-cadherin was remarkably increased to about 8-fold when siZEB1_CDS was transfected into PC-3 cells (Figure 2D). However, no or little E-cadherin activation was observed by siRNAs against the other repressors, although the expression levels of these repressors were reduced to 24–55% (Figure 2E). These results suggest that the ZEB1 is one of the potential candidates to be repressed by the seed-dependent silencing effects of dsEcad215, dsEcad302, and dsEcad640, and lead to E-cadherin activation in PC-3 cells.

### Identification of functional seed-complementary sites of dsEcad215, dsEcad302, and dsEcad640 in ZEB1 mRNA

**Figure 3:**
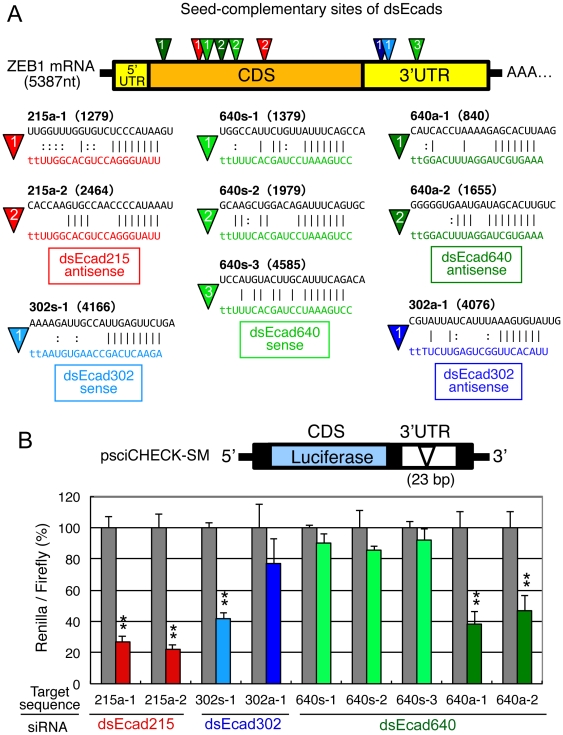
The seed-complementary sites of dsEcads in ZEB1 mRNA and silencing efficiencies of dsEcads against seed-complementary regions. (A) The seed complementary sites in the ZEB1 mRNA. In the upper panel, the sites complementary to the seed sequences of dsEcad215 antisense strand (215a-1 and -2) are indicated by red inversed triangles, that of the dsEcad302 sense strand (302s-1) by light blue, that of the dsEcad302 antisense strand (302a-1) by dark blue, those of the dsEcad640 sense strand (640s-1 to -3) by light green, and the dsEcad640a antisense strand (640a-1 and -2) by dark green. The numeral in each inversed triangle indicates the serial number of the seed complementary site of each dsEcad counting from the 5′ end of the ZEB1 mRNA. In the lower sequences, the black sequences indicate the seed-complementary target sequences in ZEB1 mRNA. The left numbers in the inverted triangles correspond to those shown on ZEB1 mRNA in the upper panel. The solid lines, Watson–Crick base pairing. Dotted lines, G∶U wobble pairing. The numerals in parentheses indicate the corresponding positions to 5′ end of each siRNA in ZEB1 mRNA, counting from the 5′end of the transcriptional start site (+1). (B) The silencing activity against each seed-complementary site. The upper panel indicates the structure of psiCHECK-SM containing 23-bp ZEB1 mRNA region with seed-complementary sequence. The lower panel shows the relative luciferase activity (*Renilla* luciferase activity/firefly luciferase activity) by the transfection of dsEcad215, dsEcad302, and dsEcad640. P-values were determined by Student's t-test (▪; P<0.01, *; P<0.05).

In ZEB1 mRNA, perfect complementary sequences to dsEcad215, dsEcad302, and dsEcad640 were not found. As shown in Table S1 and Figure 3A, 2 seed-complementary sites (215a-1 and 215a-2) of dsEcad215 antisense strand, each one seed-complementary site of dsEcad302 sense strand (302s-1) and its antisense strand (302a-1), 3 sites of dsEcad640 sense strand (640s-1 and 640s-2), and 2 sites of dsEcad640 antisense strand (640a-1 and 640a-2) were found in the ZEB1 mRNA. Among them, 215a-1 and 215a-2, 640s-1 and 640s-2, and 640a-1 and 640a-2 were situated in CDS, and 302s-1, 302a-1, and 640s-3 were in the ZEB1 3′ untranslated region (UTR).

The silencing activities of dsEcad215, dsEcad302, and dsEcad640 against each of seed-complementary sequences were analyzed using psiCHECK-SM reporter system. Twenty-three oligonucleotides containing seed-complementary regions in ZEB1 mRNA were inserted into the 3′UTR of the *Renilla* luciferase gene in psiCHECK-1 (Figure 3B). Each of these constructs and pGL3-Control, which expresses firefly luciferase used as an internal control, were transfected into HEK293 cells along with each of dsEcads. One day after transfection, the relative luciferase activities (*Renilla* luciferase activity/firefly luciferase activity) were measured (Figure 3B). The significant silencing effects of the antisense strands of dsEcad215 and dsEcad640 were observed. In both cases, the silencing activities against two different seed-complementary sites (215a-1 and 215a-2, or 640a-1 and 640a-2) were similar regardless of different sequences in non-seed regions. The silencing activity of sense strand of dsEcad302 was also significantly detected. The antisense strand of dsEcad302 showed very weak and insignificant effect, and little or no activity was detected by dsEcad640 sense strand. These results indicated that functional RNA strands of dsEcads are dsEcad215 antisense strand, dsEcad302 sense strand, and dsEcad640 antisense strand. Their silencing efficiencies may simply reflect the thermodynamic stabilities in siRNA seed duplexes that determine the efficiencies of seed-dependent off-target effects of siRNAs [28], (see Figure 1 and Discussion).

**Figure 4:**
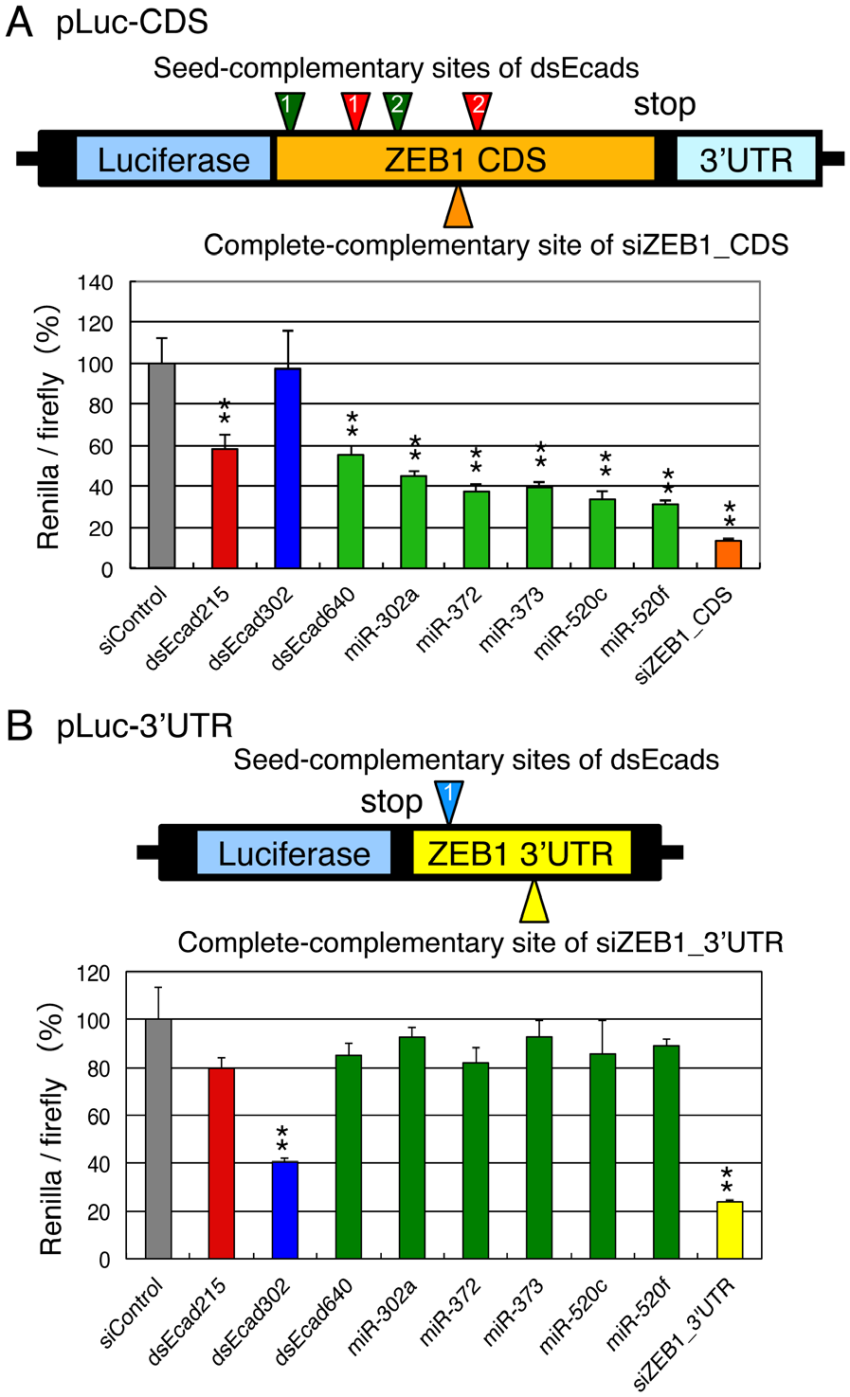
Reporter analyses using pLuc-CDS and pLuc-3′UTR. (A) Structure of pLuc-CDS. Two seed-complementary sites of the dsEcad215 antisense strand and dsEcad640 antisense strand are shown by red and dark green inverted triangles, respectively. Orange triangle indicates the complete-complementary target site of siZEB1_CDS. (B) Structure of the pLuc-3′UTR. Seed-complementary site of dsEcad302 sense strand is shown by light blue inverted triangle. Yellow triangle indicates the complete-complementary site of siZEB1_3′UTR. The silencing activities by the transfection of each siRNA and miRNA are shown as *Renilla* luciferase activity/firefly luciferase activities. The numerals in triangles show the corresponding sites shown in Figure 3. P-values were determined by Student's t-test (▪; P<0.01, *; P<0.05).

Usually, seed-dependent off-target effects are observed in mRNAs which have seed-complementary sites in their 3′UTRs. However, the seed-complementary sites of the antisense strands of dsEcad215 and the dsEcad640 were found in the ZEB1 CDS (Figure 4A). Therefore, we examined whether the repression of ZEB1 by dsEcad215 and dsEcad640 was indeed the result of targeting the CDS in the full-length context by luciferase reporter assays. The pLuc-CDS reporter, in which the CDS of ZEB1 was expressed as a fusion protein with *Renilla* luciferase gene (Figure 4A), and pLuc-3′UTR reporter, in which ZEB1 3′UTR was inserted into the 3′UTR of *Renilla* luciferase gene in psiCHECK-1 vector, were constructed (Figure 4B). pLuc-CDS or pLuc-3′UTR, and pGL3-Control were transfected into HEK293 cells with dsEcad215, dsEcad302, or dsEcad640, and the luciferase activities were measured at 1 day after transfection. Both dsEcad215 and dsEcad640 suppressed the expression of *Renilla* luciferase activities in pLuc-CDS significantly to about 60%, but little effect was observed by dsEcad302, which has no seed-complementary site in the CDS (Figure 4A). In contrast, dsEcad302, which has functional seed-complementary sites in the 3′UTR, reduced luciferase activity of the pLuc-3′UTR significantly to about 40%, whereas dsEcad215 and dsEcad640 showed little or no silencing activities (Figure 4B). These results suggest that dsEcad215 and dsEcad640 have the capabilities to reduce ZEB1 expression by acting on the seed-matched sequences in the CDS as targets, whereas dsEcad302 acts on ZEB1 3′UTR.

**Figure 5:**
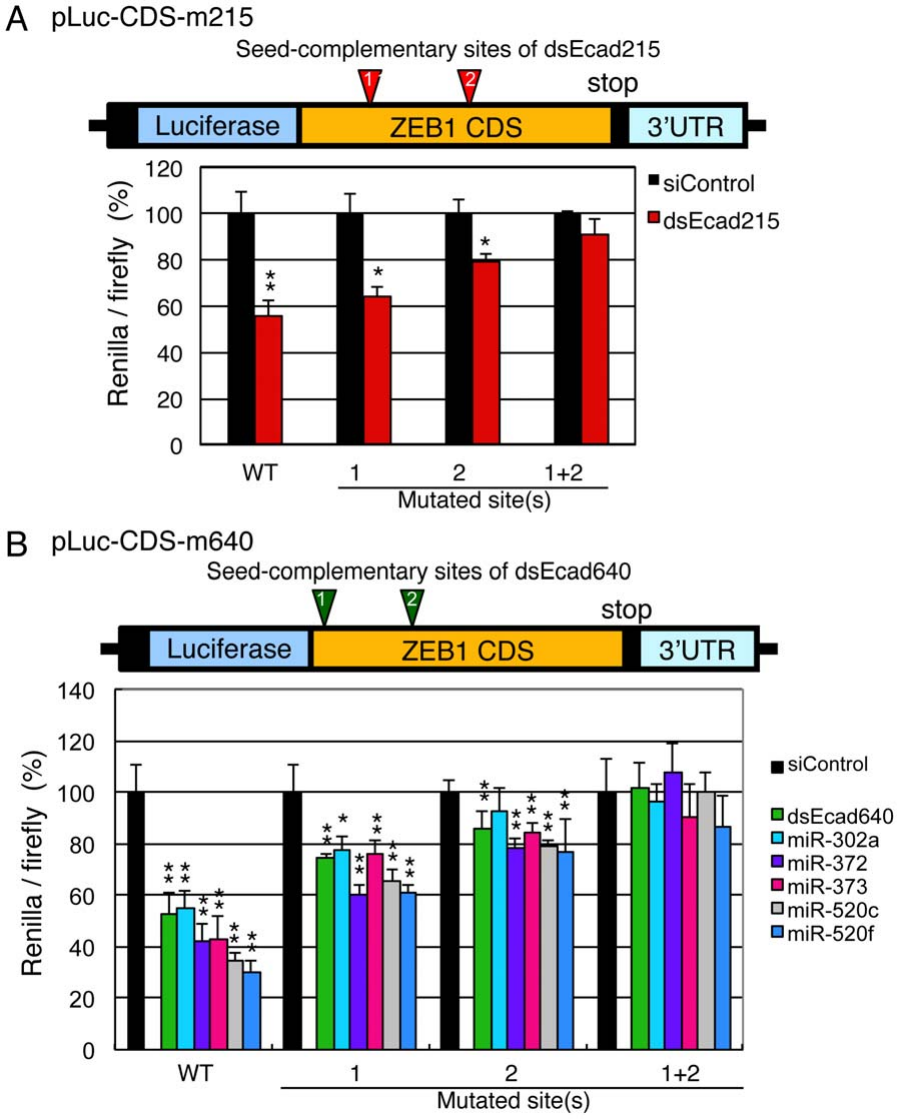
Reporter analyses of pLuc-CDS containing mutations in the seed-complementary sites. (A) Structure of the pLuc-CDS, and two seed-complementary sites of dsEcad215 antisense strand. The silencing activities by the transfection of dsEcad215 on wild-type pLuc-CDS (WT), pLuc-CDS-m215-1, -2, and -1+2 are shown as *Renilla*/firefly. (B) Structure of the pLuc-CDS and two seed-complementary sites of the dsEcad640 antisense strand. The silencing activities by dsEcad640, miR-302a, miR-372, miR-373, miR-520c, and miR-520f on wild-type pLuc-CDS (WT), pLuc-CDS-m640-1, -2, and -1+2 are shown as *Renilla*/firefly.

To confirm that the target sites of dsEcad215 and dsEcad640 are their seed-complementary sites in ZEB1 CDS, the pLuc-CDS with mutations in each of or all of the seed-complementary sites were constructed (pLuc-CDS-m215 and pLuc-CDS-m640). The pLuc-CDS with mutations in each one of two seed-complementary sites of dsEcad215 antisense strand (pLuc-CDS-m215-1 and pLuc-CDS-m215-2) were transfected with dsEcad215 along with control pGL3-Control, the relative luciferase activities were significantly reduced but slightly weak (64 and 79%, respectively) compared to the result of wild-type pLuc-CDS (56%). However, the silencing activity was almost completely eliminated when pLuc-CDS with mutations in both sites (pLuc-CDS-m215-1+2) was used (Figure 5A). Similarly, the apparent silencing activities by dsEcad640 were observed when each of two seed-complementary sites of dsEcad640 in the pLuc-CDS was mutated (pLuc-CDS-m640-1 and pLuc-CDS-m640-2) at the low levels (relative luciferase activities were 77 and 86%, respectively) compared to that of wild-type pLuc-CDS (53%). However, the silencing activity of dsEcad640 was completely inhibited when pLuc-CDS with mutations in both seed-complementary sites of dsEcad640 (pLuc-CDS-m640-1+2) was used (Figure 5B). These results clearly demonstrated that the seed-complementary sites of dsEcad215 and dsEcad640 in the ZEB1 CDS function as target sites of seed-dependent silencing.

### E-cadherin promoter assay

**Figure 6:**
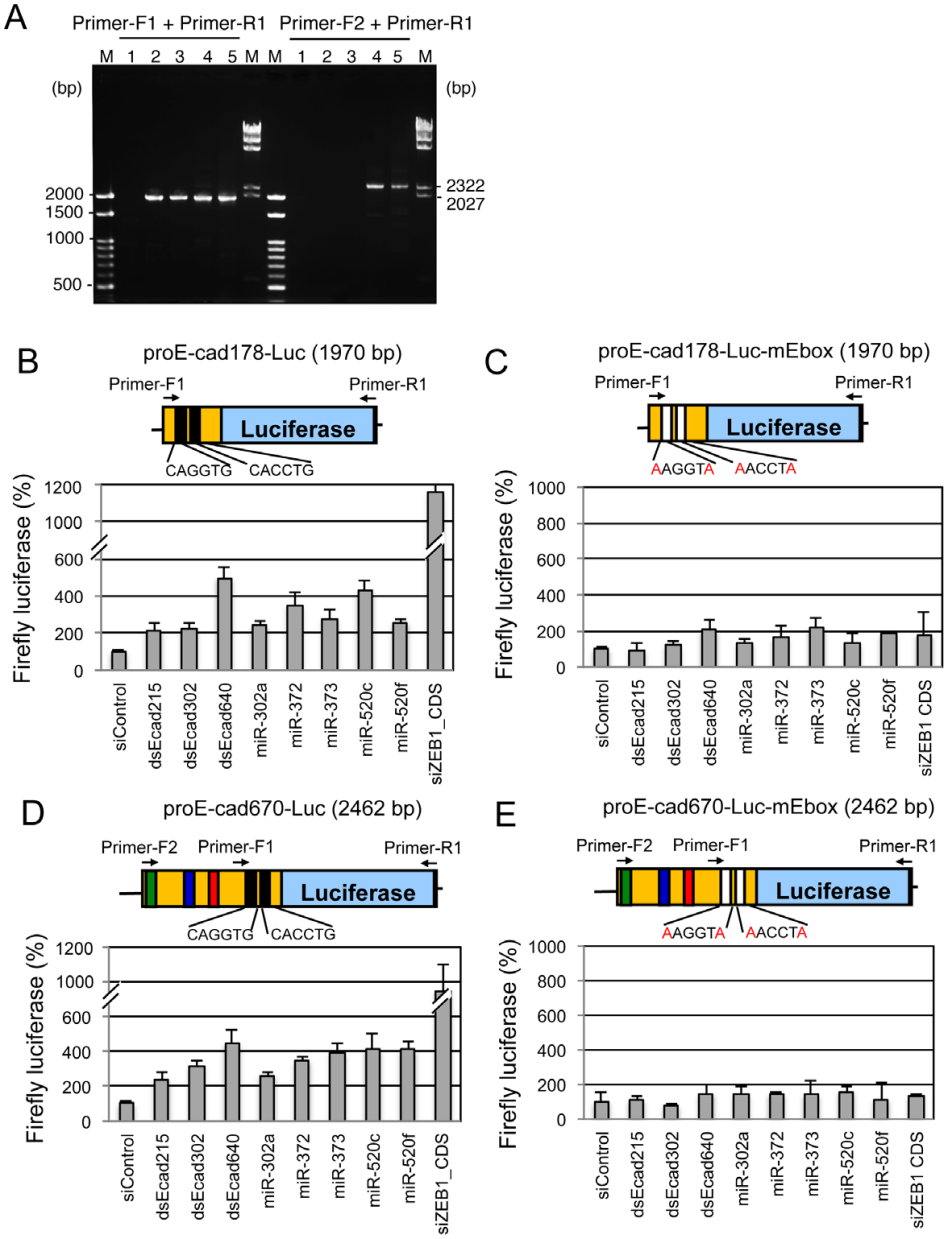
Promoter assay of E-cadherin. (A) Establishment of stable transfectants of control pGL3-Basic (lane1), proE-cad178-Luc (lane2), proE-cad178-Luc-mEbox (lane3), proE-cad670-Luc (lane4), and proE-cad670-Luc-mEbox (lane5) confirmed by genomic PCR. PCR primer set of primer-F1 and primer-R1 (see B and C) detects 1970 bp of proE-cad178-Luc, proE-cad178-Luc-mEbox, proE-cad670-Luc, and proE-cad670-Luc-mEbox. The primer set of primer-F2 and primer-R1 (see D and E) can detect 2462 bp of proE-cad670-Luc and proE-cad670-Luc-mEbox. (B–D) Structures of luciferase reporters containing E-cadherin promoters, and the effects of siRNA/miRNA for each reporter. Structures of proE-cad178-Luc containing the E-cadherin promoter region from −178 to +92 (B), proE-cad178-Luc-mEbox, in which the two upstream E-boxes were mutated (C), proE-cad670-Luc containing the E-cadherin promoter region from −670 to +92 (D), proE-cad670-Luc-mEbox, in which the two upstream E-boxes were mutated (E). The effects of dsEcad215, dsEcad302, dsEcad640, miR-302a, miR-372, miR-373, miR-520c, miR-520f, and siZEB1_CDS were determined using stably transfected cells with each luciferase reporter, and shown as *Renilla*/firefly. Note that perfect-complementary sites of dsEcad215, dsEcad302, and dsEcad640 were located at −215 (red), −302 (blue), and −640 bp (green), respectively in proE-cad670-Luc (D) and proE-cad670-Luc-mEbox (E) but not in proE-cad178-Luc and proE-cad178-Luc-mEbox. Black boxes indicate wild-type E-boxes, which nucleotide sequences are described below the E-boxes. White boxes represent mutated E-boxes, in which the substituted nucleotides are shown in red characters.

To examine the regulatory mechanisms of dsEcads on E-cadherin transcription, promoter analysis was carried out. We generated two types of E-cadherin promoter-driven firefly luciferase expression constructs: proE-cad178-Luc and proE-cad670-Luc. proE-cad178-Luc contains E-cadherin promoter region from −178 to +92, which is known to be a minimum region to exhibit promoter activity [41]. proE-cad670-Luc contains the region from −670 to +92, which contains complementary sites of dsEcad215, dsEcad302, and dsEcad640 at −215, −302, and −640 bp additively. Both of them contain two E-boxes, which are ZEB1-binding sites, situated at −24 bp and −74 bp [39]. Furthermore, E-box-mutated constructs were also generated by site-directed mutagenesis. E-box consensus sequence is CANNTG. The E-box sequences of CACCTG at −24 bp and CAGGTG at −74 bp were simultaneously mutated to AACCTA and AAGGTA , and designated as proE-cad178-Luc-mEbox and proE-cad670-Luc-mEbox, respectively. Then, PC-3 cell lines stably transfected with each reporter construct were established. The stable integration of these reporters was ascertained by genomic PCR using purified genome DNA (Figure 6A). At 3 days after transfection of dsEcad215, dsEcad302, dsEcad640, or siZEB1 into these cells, firefly luciferase activities were measured. In all reporter constructs, transfection of siZEB1 into the cells stably transfected with proE-cad178-Luc or proE-cad670-Luc increased luciferase activities efficiently up to approximately tenfold compared to the results of siControl (Figure 6B, and D). However, no or little increases of luciferase activities were observed in the cells containing proE-cad178-Luc-mEbox or proE-cad670-Luc-mEbox (Figure 6C, and E). Similarly, dsEcad215, dsEcad302, and dsEcad640 increased luciferase activities in the cells containing proE-cad178-Luc or proE-cad670-Luc up to 2∼4-fold (Figure 6B, and D), but the increase of luciferase activity was not observed in the E-box-mutated reporter-containing cells (Figure 6C, and E). These results suggested that dsEcad215, dsEcad302, and dsEcad640 increased E-cadherin transcription through E-box binding protein(s) with seed-complementarity. One of such candidate genes was presumed to be ZEB1.

Meanwhile, although E-boxes are mutated in proE-cad670-mEbox (Figure 6E), the perfect complementary sites of dsEcad215, dsEcad302, and dsEcad640 are exist in this reporter. No of little increase of luciferase activities by the transfection of dsEcads was observed, indicating that perfect complementary sites of dsEcads in ZEB1 promoter are not functional in our reporter assay system.

### Increase of E-cadherin expression via suppression of the ZEB1 transcription factor by miR-302/372/373/520 family miRNAs

The seed sequence of dsEcad640 antisense strand (AAGUGCU) is same as those of the miR-302/372/373/520 miRNA family members, miR-302a, miR-372, miR-373, miR-520a-3p, and miR-520f, although the seed sequence of miR-520f is shifted by 1 nt to 1–7 nt from 2–8 nt (Figure 1B). As shown above, the seed-complementary sites of dsEcad640 were found in ZEB1 CDS, and expected to regulate E-cadherin expression. Then, the effects on ZEB1 CDS were determined by the transfection of miR-302a, miR-372, miR-373, miR-520c, and miR-520f duplexes along with pLuc-CDS and pGL3-Control into PC-3 cells (Figure 1). In the similar fashion as shown by dsEcad640, the transfection of these miRNAs showed apparent silencing activities on *Renilla* luciferase gene fused with ZEB1 CDS (Figure 4A), but no or little silencing activities were observed when pLuc-3′UTR was co-transfected (Figure 4B). Furthermore, the reduction of relative luciferase activities by the transfection of these miRNAs with pLuc-CDS were partially repressed by introducing mutations into each of two seed-matched sites of dsEcad640 antisense strand, and the silencing activity was almost perfectly eliminated when both sites were simultaneously mutated (Figure 5B). Thus, the members of the miR-302/372/373/520 miRNA family were revealed to be able to reduce the expression of ZEB1 by targeting its CDS.

In the promoter analyses, miR-302a, miR-372, miR-373, miR-520c, and miR-520f duplexes showed the effects to increase the expression of proE-cad178-Luc and proE-cad670-Luc (Figure 6B, and D). However, no or little increases in luciferase activities were observed when the reporters with mutated E-boxes were used (Figure 6C, and E). These results suggest that members of the miR-302/372/373/520 miRNA family activate E-cadherin transcription via repression of ZEB1 transcriptional factor in an analogous fashion to dsEcad640.

### Microarray analyses of gene expression profiles by the transfection of dsEcad640 and members of miR-302/372/373/520 family miRNAs

**Figure 7:**
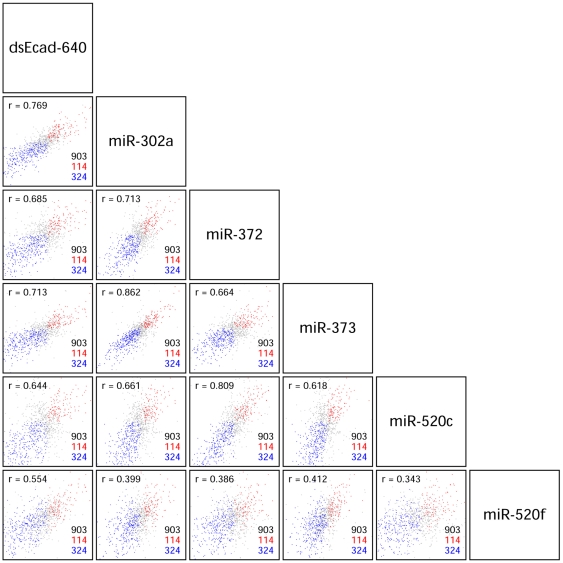
Microarray profiles of gene expression by the transfection of miR-302/372/373/520 family members and dsEcad640. In each square panel, the abscissa and longitudinal axes indicate the fold changes (log2) by the transfection of miRNAs shown in upper side and right side, respectively. The number of genes containing the common seed sequences in their 3′UTRs was 903. Among them, the number of downregulated genes was 324 (blue), and that of upregulated was 114 (red). The correlation coefficient (*r*) is indicated at the upper left side in each square panel.

To assess the parallel genome-wide regulation by dsEcad640 and the members of miR-302/372/373/520 family, microarray analyses were performed using PC-3 cells transfected with each of the miR-302a, miR-372, miR-373, miR-520c, and miR-520f duplexes, as well as with dsEcad640 at 24 hour. The mean expression levels of the transcripts that have common seed-complementary sequences, AGCACUU, to dsEcad640 and miR-302/372/373/520 miRNA family members in their 3′UTR were apparently reduced by the transfection with dsEcad640, miR-302a, miR-372, miR-373, and miR-520c duplexes (Figure S1). Among the 903 genes with the seed complementarities, the expression levels of 324 genes were commonly decreased (Figure 7, Table S2), while those of 114 genes increased (Figure 7, Table S2). The correlation coefficients of the expression levels of these common seed-complementary target genes were high between 0.62 and 0.86 each other (Figure 7). The increase of E-cadherin mRNA was not detected by microarray analyses using the cells at 24 hour after transfection, consistent with the result shown in Figure 2A. The seed sequences of dsEcad640 sense strand, miR-302*, the opposite strand of miR-372, miR-373*, miR-520c-5p, and the opposite strand of miR-520f, are different (Figure 1B). Microarray profiles showed that these variable seed sequences of the opposite strands exhibit no or little silencing activities (Figure S2). These results indicate that miR-302/372/373/520 family members and dsEcad640 show analogous genome-wide gene regulation due to the common seed sequence. The complete dataset is available at the NCBI GEO database (http://www.ncbi.nlm.nih.gov/projects/geo, accession number GSE32999).

The microarray profile of miR-520f showed relatively low correlation coefficients with the other miRNAs ranging from 0.34 to 0.41, although its correlation coefficient with dsEcad640 was 0.55 (Figure 7). The downregulated levels of transcripts with the common seed-complementarities were low (*P*≤10^−14^); however, those with own seed sequence-complementary sequence, AAGCACU, was high (*P*≤10^−57^), indicating that the target recognition of miR-520f is slightly fluctuated.

To confirm the reliability of the microarray data, we analyzed total of 48 transcripts, 8 transcripts from each of 6 transfectants, by qRT-PCR (Figure S3). The expression levels estimated by qRT-PCR were essentially identical to those obtained in the microarray analysis, with an estimated correlation coefficient of 0.89.

## Discussion

**Figure 8:**
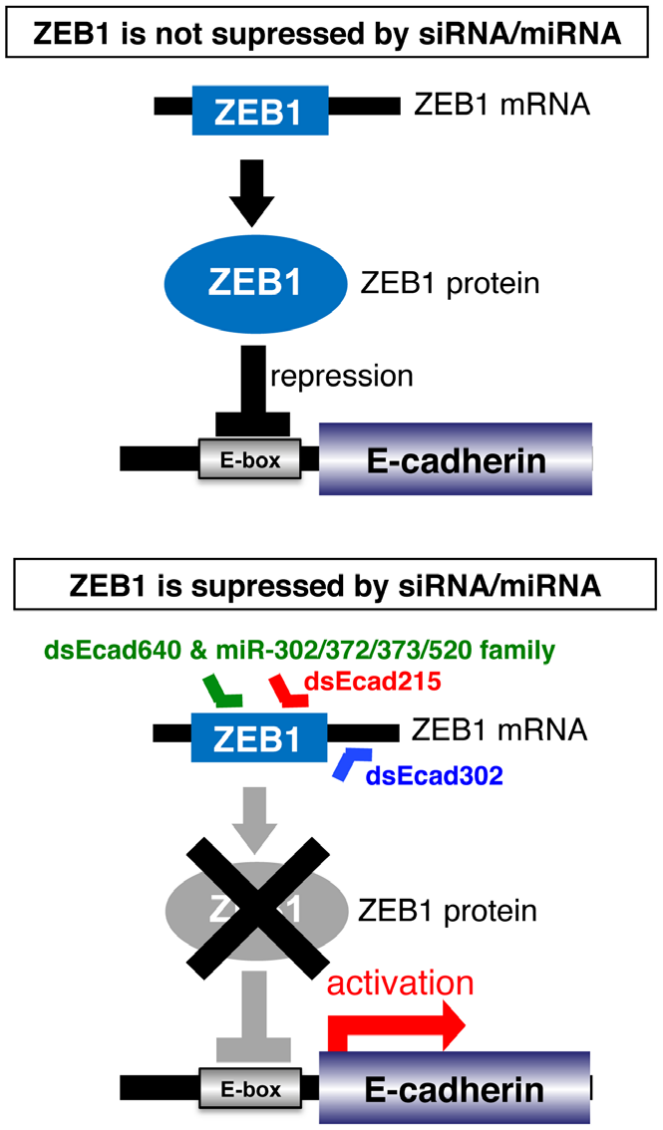
Possible mechanism of E-cadherin activation by small RNAs. The transcription of E-cadherin is suppressed by ZEB1 transcriptional repressor in PC-3 cells (upper panel). However, when dsEcad215, dsEcad302, dsEcad640, or members of miR-302/372/373/520 family act on ZEB1 mRNA and suppress the expression of ZEB1, the transcriptional repression of E-cadherin is alleviated (lower panel).

E-cadherin is reported to be transcriptionally activated by the dsEcad215, dsEcad302, and dsEcad640 by targeting to the promoter directly [30], [36]. However, in this study, we revealed that E-cadherin is also activated by these dsEcads as a result of the suppression of ZEB1 transcriptional repressor by siRNA seed-dependent off-target effects (Figure 8) based on the following results: (1) dsEcad215, dsEcad302, and dsEcad640 reduced the expression of the ZEB1 transcriptional repressor (Figure 2); (2) the functional seed-complementary sites of dsEcad215 and dsEcad640 were observed in the CDS of the ZEB1 mRNA, and those of dsEcad302 were found in the 3′UTR (Figure 3); (3) reporter analyses indicated that seed-complementary sites in the CDS function as seed-dependent silencing sites of dsEcad215 and dsEcad640, and that in the 3′UTR functions as a target site of dsEcad302 (Figures 4 and 5); (4) E-boxes in the E-cadherin promoter were indispensable for activation of E-cadherin by dsEcad215, dsEcad302, and dsEcad640 in our reporter assays (Figure 6). In our study, dsEcad215, dsEcad302, and dsEcad640 could not enhance the transcription of luciferase reporter gene, proE-cad670-Luc-mEbox, containing their perfect-complementary sites (Figure 6E), indicating that promoter-directed RNAa was not represented in our reporter analyses. However, our result might demonstrate discriminate seed-dependent function of siRNAs from their direct function on E-cadherin promoter, since participation of the region other than −670 to +92 could not be evaluated in our promoter assay. Thus, RNAa and siRNA seed-dependent silencing effect might be induced simultaneously but independently.

It is likely that transcriptional regulators other than ZEB1 can play the equivalent role in transcriptional activation of endogenous E-cadherin as shown in this study. For example, ZEB2 is also known to be a transcriptional repressor of E-cadherin. ZEB2 represses E-cadherin transcription by binding to E-boxes [40], and has 4 seed-complementary sites of dsEcad215 antisense strand, 2 sites of dsEcad302 sense strands, 1 site of dsEcad640 sense strand, and 1 site of dsEcad640 antisense strand (Table S1). In our study, the expression of ZEB2 was not detected by qRT-PCR in PC-3 cells, probably because its expression level is very low in the cells. However, if ZEB2 expression is sufficiently high, E-cadherin activation is likely to be induced via repression of ZEB2 by seed-dependent off-target effects. Thus, the transcriptional regulation by the siRNA/miRNA seed-dependent silencing effects might be different among the cell types.

We previously reported that thermodynamic stability between the siRNA seed sequence and target mRNA determines the efficiency of off-target effects [14]. The thermodynamic stability could be described as the melting temperature (T_m_) of the seed duplex; siRNA with a high seed T_m_ shows strong off-target silencing effect, while that with a low T_m_ has weak off-target activity. The seed-dependent silencing efficiencies of the siRNAs studied here are essentially correlated with the T_m_s in their seed duplexes as shown previously [14]. The calculated T_m_ in the seed duplex of dsEcad215 antisense strand is the highest at 32.1°C (Figure 1), and showed the strongest silencing effect on the target genes (Figure 3B). The seed T_m_ s are also high in dsEcad640 antisense strand (29.5°C) and dsEcad302 sense strand (26.5°C), and showed sufficient silencing activities. However, no or weak silencing activities of dsEcad302 antisense strand and dsEcad640 sense strand were detected, probably because their T_m_ s are low at 19.1°C and 12.1°C, respectively.

The seed complementary sites in the ZEB1 CDS were found to be target sites of seed-dependent silencing by dsEcad215, dsEcad640, and miR-302/372/373/520 family members (Figure 4A). Most of all the identified target sites of endogenous miRNAs are known to be located in the 3′UTRs of transcripts [13], [38], [44] with few exceptions [45]–[47]. The biological basis suggested for this observation is that ribosomal complexes override on the CDS and inhibit the miRNA-programmed RISC from attaching to the target site; when the translational process is slowed, less physical constraint is imposed by the ribosomes, thus allowing the RISC to attach to the target [48]. Because the expression level of ZEB1 is not so high in PC-3 cells, siRNA/miRNA might easily accessible to ZEB1 CDS. However, the seed-complementary sites in pLuc-CDS, which is driven by SV40 promoter, were also functional (Figure 4A). Thus, additive factor(s), such as thermodynamic stability in the seed duplex, might also account for regulation of the silencing efficiencies on CDS. The siRNA with the higher seed duplex stability might easily access to CDS compared to that with low stability.

In this study, the members of the endogenous miR-302/372/373/520 family were found to suppress ZEB1 transcription factor in PC-3 cells and finally activate the expression of E-cadherin through a similar regulatory pathway induced by dsEcad640. This regulatory pathway might be functional in some situations such as the induction of pluripotency *in vivo*. A noncoding RNA cluster containing mir-302b, mir-302c, mir-302a, and mir-367 are known to be expressed most abundantly in human ES cells, and quickly decrease after cell differentiation and proliferation [49]. The transfection of the mir-302 cluster into human Colo and PC-3 cells has been shown to generate ES-like cells, known as miRNA-induced pluripotent stem (mirPS) cells [50]. In the unrelated studies, E-cadherin expression is shown to be profoundly linked to the induction of pluripotency in bFGF, Activin, and BIO-derived mouse stem cells (FAB-SCs) [34]. FAB-SCs are blastocyst-derived stem cells, but have no potency for embryoid body and teratoma formation, or embryonic development upon blastocyst transplantation. However, brief stimulation by Leukemia Inhibitory Factor (LIF) and Bone Morphogenetic Protein 4 (BMP4) upregulates E-cadherin expression to levels comparable to those observed in mouse ES cells, and induces the pluripotent state to allow the generation of teratoma and give germ line contribution in chimeric mice. Our result might account for a pathway to gain pluripotency by linking the mirPS induction pathway by miR-302 and that to induce pluripotent state of FAB-SCs by E-cadherin upregulation.

## Materials and Methods

### Preparation of siRNAs and miRNAs

DsEcad215, dsEcad302, and dsEcad640 were chemically synthesized (Sigma). MiR-302a, miR-372, miR-373, miR-520c, miR-520f miRNA duplexes were synthesized to form the same sequences and structures described in miRBase [51]. SiRNAs against ZEB1 CDS and 3′UTR, SNAIL, SLUG, and E12/E47 were designed using our algorithms for selecting highly functional siRNAs [14], [52]. SiRNA which has no seed-complementary sequence against ZEB1, firefly and *Renilla* luciferase genes was used as siControl. MiRNA and siRNA sequences used in this study were shown in Figure 1 and Table S3.

### Construction of luciferase reporters

The silencing efficacies due to seed-complementary regions in ZEB1 mRNA were analyzed by psiCHECK-SM reporters (Figure 3B). Both strands of 23-nt oligonucleotides containing the seed-complementary regions of dsEcad215, dsEcad302, and dsEcad640 in ZEB1 mRNA with overhang sequence of either EcoRI or NotI were chemically synthesized (Table S4). After annealing them, the double-stranded oligonucleotides were inserted into the 3′UTR of the *Renilla* luciferase gene in the psiCHECK-1 vector digested with the same restriction enzymes to generate psiCHECK-SM.

The ZEB1 CDS was amplified from cDNAs synthesized from PC-3 mRNAs using primers with restriction enzyme sites of SalI and NotI on each end (Table S5). psiCHECK-1 vector was also amplified using primers with same enzyme sites. Both the amplified ZEB1 CDS and psiCHECK-1 were digested with SalI and NotI, and ligated to generate pLuc-CDS. Then, ZEB1 CDS was expressed as a fusion protein with *Renilla* luciferase in pLuc-CDS by eliminating the stop codon by using primers, pLuc-ZEB1-CDS-F and -R (Table S5). The ZEB1 3′UTR was amplified from cDNAs from PC-3 mRNAs using primers with restriction enzyme sites of EcoRI and NotI (Table S5) on each end and inserted into psiCHECK-1 digested with the same restriction enzymes to generate pLuc-3′UTR. The site-directed mutagenesis at each seed-complementary site in dsEcad215, dsEcad302, or dsEcad640 was carried out using the PCR primers shown in Table S5.

For promoter analysis, the E-cadherin promoter region from −178 to +92 and −670 to +92 were amplified using primers with restriction enzyme sites of BglII or HindIII at each end (Table S5) from genomic DNA of HeLa cells. Amplified PCR products were inserted into the upstream region of the firefly luciferase gene of the pGL3-Basic vector (Promega) and named proE-cad178-Luc and proE-cad670-Luc, respectively (see Figure 6). Two E-boxes were mutated by site-directed mutagenesis using the PCR primers shown in Table S5 and designated as E-cad178-Luc-mEbox and proE-cad670-Luc-mEbox, respectively.

The constructs were purified with the Genopure Plasmid Midi Kit (Roche) and sequenced.

### Cell culture and transfection

Human prostate cancer-derived PC-3 cells and human embryonic kidney-derived HEK293 cells were cultured in RPMI-1640 medium (Wako Pure Chemical Industries) and Dulbecco's modified Eagle's medium (Invitrogen), respectively, with 10% fetal bovine serum (Sigma) at 37°C. The day before transfection, the cells were inoculated at 5×10^4^ cells/mL/well in each well of a 24-well cell culture plate (diameter, 15 mm).

To investigate the changes in mRNA levels of E-cadherin, ZEB1, SNAIL, SLUG, and E12/E47, PC-3 cells were transfected with each dsEcad or siRNA against ZEB1, SNAIL, SLUG, or E12/E47 (5 nM) using Lipofectamine 2000 (Invitrogen) according to the manufacture's instruction. At the indicated times after transfection, mRNA levels were measured by qRT-PCR.

In the luciferase reporter assay of dsEcads and miRNAs for their seed-matched sequences in ZEB1 CDS and 3′UTR, HEK293 cells were transfected with each dsEcad or miRNA (5 nM) and pGL3-Control, which is firefly luciferase expression vector (0.5 µg; Promega) used as an internal control, along with psiCHECK-SM, pLuc-CDS, or pLuc-3′UTR (0.1 µg) simultaneously. At 1 day after transfection, firefly and *Renilla* luciferase activities were measured using the Dual Luciferase Reporter Assay System (Promega) with ARVO SX 1420 Multilabel Counter (PerkinElmer Life Sciences), and the *Renilla* luciferase activities relative to the firefly luciferase activities were calculated.

For promoter analysis, PC-3 cells stably transfected with proE-cad178-Luc, proE-cad670-Luc, proE-cad178-Luc-mEbox, or proE-cad670-Luc-mEbox were established. Each promoter-containing construct (0.5 µg) was transfected into PC-3 cells with pSilencerPuro (0.5 µg; Ambion), which expresses Puromycin resistant gene. The cells were selected with Puromycin (2 µg/ml) for about 3 weeks. Then, each dsEcad or miRNA (5 nM) was transfected into the cells. At 3 days after transfection, firefly luciferase activity was measured. Establishment of stable cell lines was confirmed by genomic PCR using primers shown in Table S6.

### Quantitative RT-PCR

Total RNA was purified using the RNeasy Mini Kit (Qiagen) according to manufacture's instruction. cDNA was synthesized by reverse transcription using the Anchored-oligo (dT) 18 primer with the Transcriptor High Fidelity cDNA Synthesis Kit (Roche). The qRT-PCR was performed with primers specific for each gene (Table S6) using the FastStart Universal SYBR Green Master (Roche) with ABI PRISM 7000 (Applied Biosystems). The reaction was carried out at 95°C for 10 min, followed by 40 cycles of 95°C for 15 seconds and 60°C for 1 min.

### Western blot

The cells transfected with dsEcads were recovered with Passive Lysis Buffer (Promega). Each of 5 µg of total protein was mixed with the equal volume of 2× SDS PAGE Sample Buffer (4% SDS, 0.1 M Tris-HCl (pH 6.8), 12% 2-mercaptoethanol, 20% glycerol, 0.01% bromophenol blue). After boiling for five minutes, the lysate was separated by SDS-PAGE and transferred to the BioTraceTM PVDF membrane (Pall Corporation) using iBrotTM Dry Blotting System (Invitrogen). The membrane was blocked for 1 hour in TBS-T (20 mM Tris-HCl (pH 7.5), 150 mM NaCl, 0.2% Triton X-100) supplemented with 5% DifcoTM Skim Milk (Becton, Dickinson and Company), and incubated with ×1,000-diluted anti-ZEB1 antibody (SANTA CRUZ BIOTECHNOLOGY, INC.) or ×2,000-diluted anti-GAPDH antibody (Ambion) at 4°C overnight. The membrane was washed three times with TBS-T, and reacted with ×10,000-diluted alkaline phosphatase-labeled anti-rabbit or anti-mouse antibody (Cappel) at 4°C overnight. After being washed three times with TBS-T, the membrane was transferred into alkaline phosphatase development solution (100 mM Tris-HCl (pH 9.5), 100 mM NaCl, 5 mM MgCl_2_), and reacted with CDP-StarTM detection reagent (GE Healthcare), and visualized with LAS3000 (FUJIFILM).

### Microarray analysis

Chemically synthesized dsEcad640, miR-302a, miR-372, miR-373, miR-520c, and miR-520f duplexes were transfected into PC-3 cells. At 24 hours after transfection, the total RNA was extracted using the RNeasy Mini Kit. As a control, mock transfected cells treated with transfection reagent without siRNA and miRNA were used. Transfection was carried out four times independently, and the RNA quality was examined using Bioanalyzer (Agilent). Mixed RNA samples (150 ng total) of four experiments were used for the microarray analysis. Cy-3 labeled cRNA was prepared using the Quick Amp Labeling Kit (Agilent), and purified with the RNeasy Mini Kit. The cRNA was hybridized against the Agilent Whole Human Genome Microarray (4×44 K multipack format) at 65°C for 17 hours, then washed with Gene Expression Wash Buffer 1 and Wash Buffer 2 (Agilent) in 0.005% Triton X-102. The microarray slide was scanned using the DNA Microarray Scanner (Agilent) and quantified using Feature Extraction Software (Agilent). To confirm the results of the microarray, qRT-PCR was carried out using the PCR primers shown in Table S6.

### Accession number

The microarray data reported herein are available at the NCBI GEO database (http://www.ncbi.nlm.nih.gov/projects/geo, accession number GSE32999).

### Statistical analysis

Differences of significance in the expression levels of mRNAs and luciferase activities of reporter analyses were analyzed by the unpaired Student's t-test. Microarray data were analyzed by the one-sided Kolmogorov–Smirnov (K-S) test.

## Supporting Information

Figure S1 **Microarray profiles of transcripts containing common seed-complementary sequences in their 3′UTRs.** Microarray profiles of transcripts containing common seed-complementary sequences of dsEcad640 and members of miR-302/372/373/520 family by the transfection of (A) dsEcad640, (B) miR-302a duplex, (C) miR-372 duplex, (D) miR-373 duplex, (E) miR-520c duplex, and (F) miR-520f duplex. The left panels represent MA plots. The changes in gene expression are shown as log2 of the fold change ratio (ordinate), relative to mock transfection. The abscissa is the signal intensity of the transcript (log2 scale). Red and gray dots, respectively, represent transcripts complementary to the seed sequences and those with no seed complementarity. The right panels indicate the cumulative fraction of transcripts with one or more sequences complementary to the seed sequences of each siRNA and miRNA. The red and gray lines indicate the cumulative fraction of transcripts with and without seed complementarity, respectively. Results of a one-sided K-S test for seed-dependent off-target effects are as follows: transcripts with seed-complementary sequences of dsEcad640, P≤10^−59^; those of miR-302a, P≤10^−45^; those of miR-372, P≤10^−20^; those of miR-373, P≤10^−39^; those of miR-520c, P≤10^−40^; those of miR-520f using common seed sequence, P≤10^−14^; miR-520f using own seed sequence, P≤10^−57^.
(TIF)

Figure S2 **Microarray profiles of transcripts containing variable seed-complementary sequences in their 3′UTRs.** Microarray profiles of transcripts containing variable seed-complementary sequences of the opposite strands in their 3′UTRs by the transfection of (A) dsEcad640, (B) miR-302a duplex, (C) miR-372 duplex, (D) miR-373 duplex, (E) miR-520c duplex, and (F) miR-520f duplex. The left panels show MA plots. Blue and gray dots, respectively, represent transcripts complementary to the seed of the opposite strands and those with no seed complementarity. The right panels indicate the cumulative fraction of transcripts with one or more sequences complementary to the opposite strand seed sequences of each siRNA and miRNA. The blue line indicates the cumulative fraction of transcripts with one or more sequences complementary to the siRNA and miRNA guide strand seeds. The gray line shows transcripts with no seed complementarity. Results of a one-sided K-S test for seed-dependent off-target effects is as follows: transcripts with complementary seed sequences of the opposite strand of dsEcad640, P = 0.999; those of miR-302a, P = 0.266; those of miR-372, P = 0.449; those of miR-373, P = 0.953; those of miR-520c, P = 0.031; those of miR-520f, P = 0.998. Note that no significant silencing effects were detected for transcripts with seed-complementary sequences of the opposite strands.
(TIF)

Figure S3 **Comparison of microarray data with those of qRT-PCR.** (A) Eight genes (ZEB1, MED8, MTPN, LATS2, RAB31, GAPDH, HINT1, PLEKHC1) were arbitrarily chosen, and changes in mRNA expression level compared with mock transfection were examined by qRT-PCR (abscissa) and microarray (ordinate). Note that the results of microarray are almost linearly correlated with those of qRT-PCR. The correlation coefficient was estimated at 0.89. Comparison at the level of individual gene is shown in (B–I); (B) ZEB1, (C) MED8, (D) MTPN, (E) LATS2, (F) RAB31, (G) GAPDH, (H) HINT1, and (I) PLEKHC1. The transcripts of ZEB1, MED8, MTPN, LATS2, and RAB31 possess seed-complementarities to either of dsEcad640, miR-302a, miR-372, miR-373, miR-520c, and miR-520f.
(TIF)

Table S1 **Complementary sites of dsEcad215, dsEcad302, and dsEcad640 seed regions in the negative regulators of E-cadherin.**
(PDF)

Table S2 **List of the increased and decreased genes that have common seed-complementary sequences to dsEcad640 and miR-302/372/373/520 miRNA family members.**
(XLS)

Table S3 **SiRNA sequences used in this study.**
(PDF)

Table S4 **Oligonucleotides for construction of psiCHECK-SM.**
(PDF)

Table S5 **Oligonucleotides for construction of pLuc-CDS, pLuc-3′UTR, proE-cad-178-Luc, and proE-cad670-Luc reporters, and site-directed mutagenesis of seed-complementary sites of dsEcad215 and dsEcad640 and E-boxes in E-cadherin promoters.**
(PDF)

Table S6 **PCR primers used in this study.**
(PDF)
