# Supplementary figures and images for: E-Cadherin Is Transcriptionally Activated via Suppression of ZEB1 Transcriptional Repressor by Small RNA-Mediated Gene Silencing
Source: PLoS One. 2011 Dec 21;6(12):e28688. doi: 10.1371/journal.pone.0028688 (PMC3244408; doi:10.1371/journal.pone.0028688)

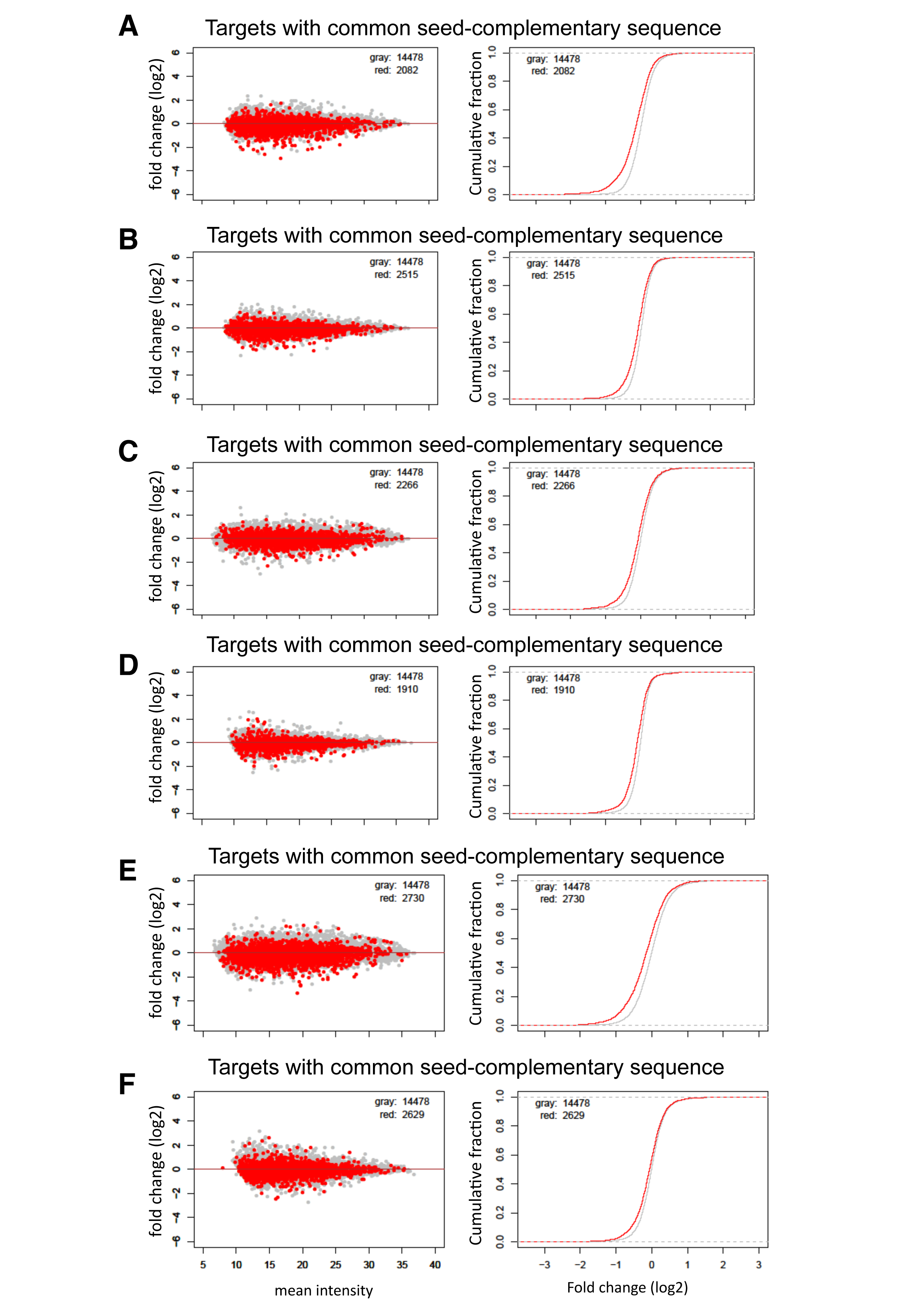

Supplement: Figure S1 — Microarray profiles of transcripts containing common seed-complementary sequences in their 3′UTRs. Microarray profiles of transcripts containing common seed-complementary sequences of dsEcad640 and members of miR-302/372/373/520 family by the transfection of (A) dsEcad640, (B) miR-302a duplex, (C) miR-372 duplex, (D) miR-373 duplex, (E) miR-520c duplex, and (F) miR-520f duplex. The left panels represent MA plots. The changes in gene expression are shown as log2 of the fold change ratio (ordinate), relative to mock transfection. The abscissa is the signal intensity of the transcript (log2 scale). Red and gray dots, respectively, represent transcripts complementary to the seed sequences and those with no seed complementarity. The right panels indicate the cumulative fraction of transcripts with one or more sequences complementary to the seed sequences of each siRNA and miRNA. The red and gray lines indicate the cumulative fraction of transcripts with and without seed complementarity, respectively. Results of a one-sided K-S test for seed-dependent off-target effects are as follows: transcripts with seed-complementary sequences of dsEcad640, P≤10−59; those of miR-302a, P≤10−45; those of miR-372, P≤10−20; those of miR-373, P≤10−39; those of miR-520c, P≤10−40; those of miR-520f using common seed sequence, P≤10−14; miR-520f using own seed sequence, P≤10−57. (TIF) [file pone.0028688.s001.tif]

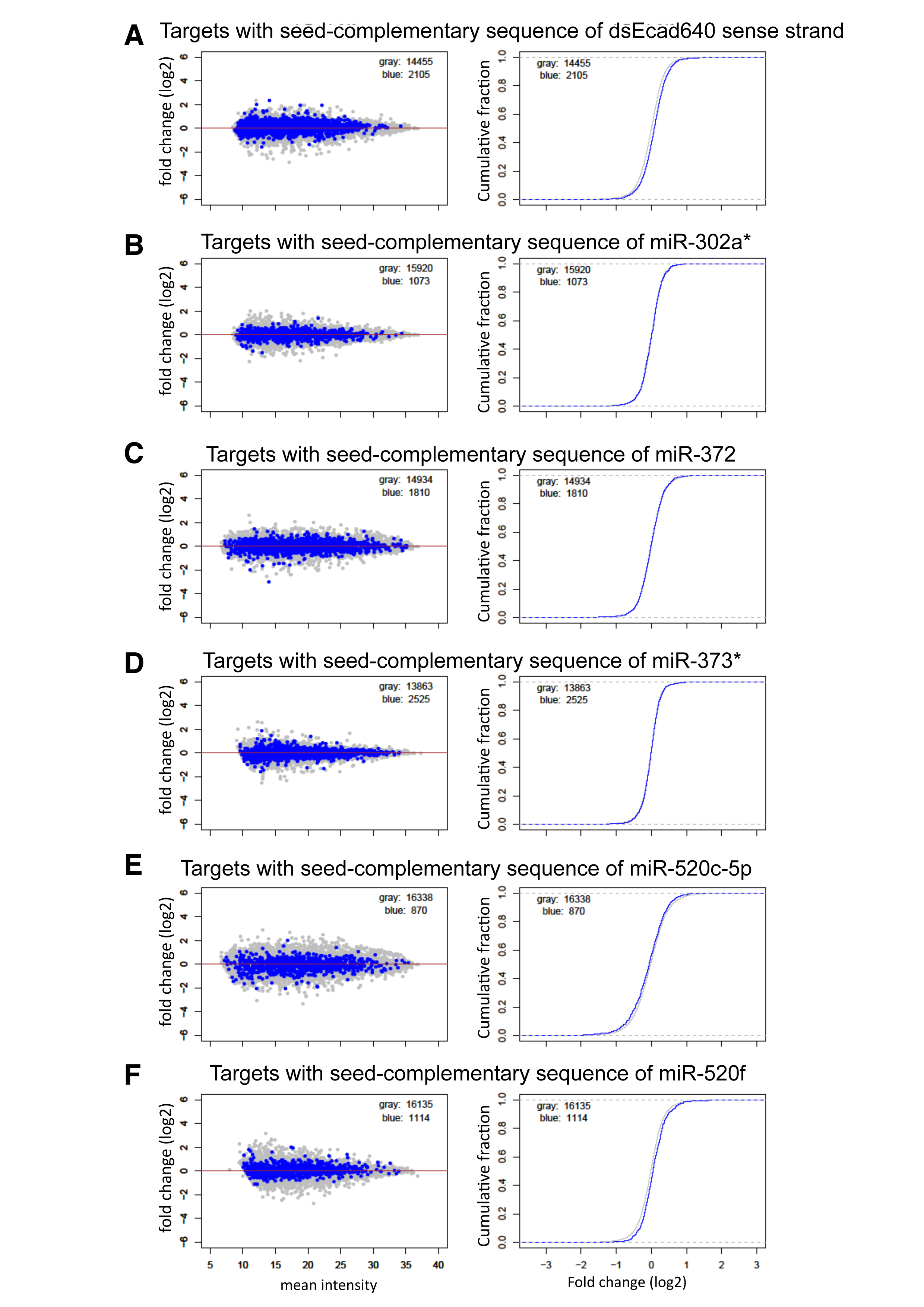

Supplement: Figure S2 — Microarray profiles of transcripts containing variable seed-complementary sequences in their 3′UTRs. Microarray profiles of transcripts containing variable seed-complementary sequences of the opposite strands in their 3′UTRs by the transfection of (A) dsEcad640, (B) miR-302a duplex, (C) miR-372 duplex, (D) miR-373 duplex, (E) miR-520c duplex, and (F) miR-520f duplex. The left panels show MA plots. Blue and gray dots, respectively, represent transcripts complementary to the seed of the opposite strands and those with no seed complementarity. The right panels indicate the cumulative fraction of transcripts with one or more sequences complementary to the opposite strand seed sequences of each siRNA and miRNA. The blue line indicates the cumulative fraction of transcripts with one or more sequences complementary to the siRNA and miRNA guide strand seeds. The gray line shows transcripts with no seed complementarity. Results of a one-sided K-S test for seed-dependent off-target effects is as follows: transcripts with complementary seed sequences of the opposite strand of dsEcad640, P = 0.999; those of miR-302a, P = 0.266; those of miR-372, P = 0.449; those of miR-373, P = 0.953; those of miR-520c, P = 0.031; those of miR-520f, P = 0.998. Note that no significant silencing effects were detected for transcripts with seed-complementary sequences of the opposite strands. (TIF) [file pone.0028688.s002.tif]

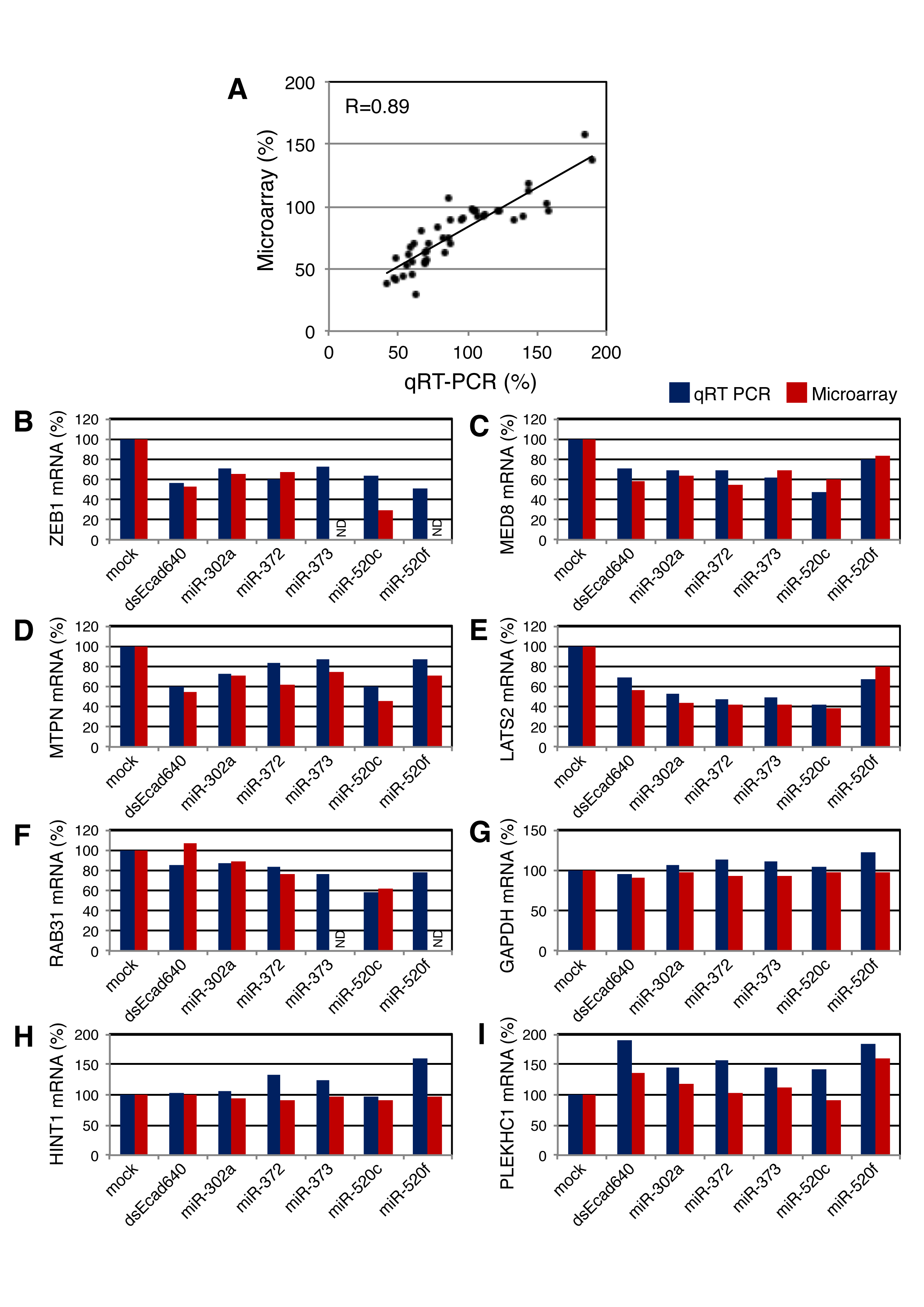

Supplement: Figure S3 — Comparison of microarray data with those of qRT-PCR. (A) Eight genes (ZEB1, MED8, MTPN, LATS2, RAB31, GAPDH, HINT1, PLEKHC1) were arbitrarily chosen, and changes in mRNA expression level compared with mock transfection were examined by qRT-PCR (abscissa) and microarray (ordinate). Note that the results of microarray are almost linearly correlated with those of qRT-PCR. The correlation coefficient was estimated at 0.89. Comparison at the level of individual gene is shown in (B–I); (B) ZEB1, (C) MED8, (D) MTPN, (E) LATS2, (F) RAB31, (G) GAPDH, (H) HINT1, and (I) PLEKHC1. The transcripts of ZEB1, MED8, MTPN, LATS2, and RAB31 possess seed-complementarities to either of dsEcad640, miR-302a, miR-372, miR-373, miR-520c, and miR-520f. (TIF) [file pone.0028688.s003.tif]
