# Supplementary material for: E-Cadherin Is Transcriptionally Activated via Suppression of ZEB1 Transcriptional Repressor by Small RNA-Mediated Gene Silencing
Source: PLoS One. 2011 Dec 21;6(12):e28688. doi: 10.1371/journal.pone.0028688 (PMC3244408; doi:10.1371/journal.pone.0028688)
Supplement: Table S1 — Complementary sites of dsEcad215, dsEcad302, and dsEcad640 seed regions in the negative regulators of E-cadherin. (PDF) [file pone.0028688.s004.pdf]

Table S1

Complementary sites of dsEcad215, dsEcad302, and dsEcad640 seed regions in the negative regulators of E-cadherin.

Seed-complementary sites of dsEcad215

Gene name	Accession No.	Full-length mRNA	CDS	RNA strand	No. of seed-complementary sites		Potision corresponding to 5'end of each strand of dsEcad215		
					CDS	3'UTR			
ZEB1	NM_030751	1..5387	64..3438	sense	0	0			
				antisense	2	0	1279	2464	
ZEB2	NM_014795	1..924	523..4167	sense	0	0			
				antisense	1	0	7212		
SNAIL	NM_005985	1..1722	85..879	sense	0	0			
				antisense	0	0			
SLUG	NM_003068	1..2112	176..982	sense	0	0			
				antisense	0	1	1393		
E12/E47	NM_003200	1..4462	68..2032	sense	0	0			
				antisense	0	0			

Seed-complementaryt sites of dsEcad302

Gene nam	Accession No.	Full-length mRNA	CDS	RNA strand	No. of seed-complementary sites		Potision corresponding to 5'end of each strand of dsEcad302		
					CDS	3'UTR			
ZEB1	NM_030751	1..5387	64..3438	sense	0	1	4166		
				antisense	0	1	4076		
ZEB2	NM_014795	1..924	523..4167	sense	0	2	5622	8431	
				antisense	0	0			
SNAIL	NM_005985	1..1722	85..879	sense	0	0			
				antisense	0	0			
SLUG	NM_003068	1..2112	176..982	sense	0	1	1897		
				antisense	0	0			
E12/E47	NM_003200	1..4462	68..2032	sense	0	0			
				antisense	0	0			

Seed-complementary sites of dsEcad640

Gene nam	Accession No.	Full-length mRNA	CDS	RNA strand	No. of seed-complementary sites		Potision corresponding to 5'end of each strand of dsEcad640		
					CDS	3'UTR			
ZEB1	NM_030751	1..5387	64..3438	sense	2	1	1379	1979	4585
				antisense	2	0	840	1655	
ZEB2	NM_014795	1..924	523..4167	sense	1	0	3409		
				antisense	1	0	1839		
SNAIL	NM_005985	1..1722	85..879	sense	0	1	1415		
				antisense	0	0			
SLUG	NM_003068	1..2112	176..982	sense	0	0			
				antisense	0	0			
E12/E47	NM_003200	1..4462	68..2032	sense	0	1	4382		
				antisense	0	0			

Seed-complementary sites were assigned by the complementarity of nucleitides positioned 2-8 and 1-7
