# Supplementary material for: E-Cadherin Is Transcriptionally Activated via Suppression of ZEB1 Transcriptional Repressor by Small RNA-Mediated Gene Silencing
Source: PLoS One. 2011 Dec 21;6(12):e28688. doi: 10.1371/journal.pone.0028688 (PMC3244408; doi:10.1371/journal.pone.0028688)
Supplement: Table S3 — SiRNA sequences used in this study. (PDF) [file pone.0028688.s006.pdf]

Table S3

SiRNA sequences used in this study.

Guide strand	Sequence (5′→3′)	Passenger strand	Sequence (5′→3′)
siZEB1-A	UGUAACGUUAUUGCGCCGCGG	siZEB1-S	GCGGCGCAAUAACGUUACAAA
siZEB1_3'-A	GCGUAUUAUCAUUUAAAGUGU	siZEB1_3'-S	ACUUUAAAUGAUAAUACGCUC
siZEB2-A	UCGUUUCGGGAUCCGUAUCCA	sZEB2-S	GAUACGGAUCCCGAAACGAUA
sSNAIL-A	UAAACUCUGGAUUAGAGUCCU	sSNAIL-S	GACUCUAAUCCAGAGUUUACC
sSLUG-A	UCUAAUGUGUCCUUGAAGCAA	sSLUG-S	GCUUCAAGGACACAUUAGAAC
sE12/E47-A	AGUUAUUGCUUGAGUGAUCCG	sE12/E47-S	GAUCACUCAAGCAAUAACUUC
siControl	GUUAGUAGUCACACUUAUATT	siControl	UAUAAGUGUGACUACUAACTT

A indicates antisense strand, S, sense strand.
