# Supplementary material for: E-Cadherin Is Transcriptionally Activated via Suppression of ZEB1 Transcriptional Repressor by Small RNA-Mediated Gene Silencing
Source: PLoS One. 2011 Dec 21;6(12):e28688. doi: 10.1371/journal.pone.0028688 (PMC3244408; doi:10.1371/journal.pone.0028688)
Supplement: Table S4 — Oligonucleotides for construction of psiCHECK-SM. (PDF) [file pone.0028688.s007.pdf]

Table S4

Oligonucleotides for construction of psiCHECK-SM.

Oligonucleotide	Sequence (5´→3´)	Oligonucleotide	Sequence (5´→3´)
215a-1-S	AATTTTGGTTTGGTGTCTCCCATAAGT	215a-1-A	GGCCACTTATGGGAGACACCAAACCAA
215a-2-S	AATTCACCAAGTGCCAACCCCATAAAT	215a-2-A	GGCCATTTATGGGGTTGGCACTTGGTG
302s-1-S	AATTAAAAGATTGCCATTGAGTTCTGA	302s-1-A	GGCCTCAGAACTCAATGGCAATCTTTT
302a-1-S	AATTCGTATTATCATTTAAAGTGTATTG	302a-1-A	GGCCCAATACACTTTAAATGATAATACG
640s-1-S	AATTTGGCCATTCTGTTATTTCAGCCA	640s-1-A	GGCCTGGCTGAAATAACAGAATGGCCA
640s-2-S	AATTGCAAGCTGGACAGATTTCAGTGC	640s-2-A	GGCCGCACTGAAATCTGTCCAGCTTGC
640s-3-S	AATTTTATGTTGATTGATTTTCAGAAT	640s-3-A	GGCCATTCTGAAAATCAATCAACATAA
640a-1-S	AATTCATCACCTAAAAGAGCACTTAAG	640a-1-A	GGCCCTTAAGTGCTCTTTTAGGTGATG
640a-2-S	AATTGGGGGTGAATGATAGCACTTGTC	640a-2-A	GGCCGACAAGTGCTATCATTCACCCCC

S indicates sense strand, A, antisense strand. Sense and antisense strand sequences are complementary. Overhang sequences of restriction enzymes are underlined. Red indicates the complementary sequence of seed region of respective miRNA.
