# Supplementary material for: E-Cadherin Is Transcriptionally Activated via Suppression of ZEB1 Transcriptional Repressor by Small RNA-Mediated Gene Silencing
Source: PLoS One. 2011 Dec 21;6(12):e28688. doi: 10.1371/journal.pone.0028688 (PMC3244408; doi:10.1371/journal.pone.0028688)
Supplement: Table S5 — Oligonucleotides for construction of pLuc-CDS, pLuc-3′UTR, proE-cad-178-Luc, and proE-cad670-Luc reporters, and site-directed mutagenesis of seed-complementary sites of dsEcad215 and dsEcad640 and E-boxes in E-cadherin promoters. (PDF) [file pone.0028688.s008.pdf]

Table S5

Oligonucleotides for construction of pLuc-CDS, pLuc-3'UTR, proE-cad-178-Luc, and proE-cad670-Luc reporters, and site-directed mutagenesis of seed-complementary sites of dsEcad215 and dsEcad640 and E-boxes in E-cadherin promoters.

Oligonucleotide	Sequence (5´→3´)
Sal1-ZEB1-CDS-F	TTTCCCGTCGACATGGCGGATGGCCCCAGGTGTAAGCGC
Not-ZEB1-CDS-R	TTTTCCTTTTGCGGCCGCTTAGGCTTCATTTGTCTTTTCTTCAG
psiCHECK1-F	TTTAAACCTAGAGCGGCCGCTGGC
Sal1-psiCHECK-R	TTTCCCGTCGACCGAATTCCCGGGCTCGAGCGATCG
pLuc-ZEB1-CDS-F	TCTAGGCGATCGCTCGAGCCCGGG
pLuc-ZEB1-CDS-R	CTGCTCGTTCTTCAGCACGCGCTC
Eco-ZEB1-3'UTR-F	TTTCCCGAATTCTCGTTTTTCTAGAAGGAAAATAAATTCTAATTG
Not1-ZEB1-3'UTR-R	TTTTCCTTTTGCGGCCGCCTTGTTGATGCATTTTATTG
215a-1-F	CCAATTTCTATCAATTTAAGTGATATTCAGAATGTACTT
215a-1-R	TGAAACCAACCCAACTGTTGGCAGAACAACAGCTTGCACCAT
215a-2-F	AACCCAATTAATATCGCTATACCTACAGTCACTGCC
215a-2-R	GGCTGATGGTGGGATTACATTTACAACTGGTTCTG
640a-1-F	AGAACATCTGAGAATTCACAGTGGAGAGAAGCCATA
640a-1-R	TTAAGGTGATGTTTGTATTTGAAAGCTTTTCCACA
640a-2-F	ACTCAACATGTCTTCTGTGTGATGATTGTCCAGGAG
640a-2-R	CGTTAACCCCCCCTTCAAAGCTTTTGTCCTTCTCA
proE-cad178-F	AAAGGGAGATCTTCCAGGCTAGAGGGTCACCGCGTC
proE-cad178-R	AAAGGGAAGCTTCCGGGTGCGGTCGGGTCGGGCCGG
proE-cad670-F	AAAGGGAGATCTGGCTGCTAGCTCAGTGGCTCATGG
proE-cad670-R	AAAGGGAAGCTTCCGGGTGCGGTCGGGTCGGGCCGG
E-box(-24)-F	AACCTAGCTGCAGCCACGCACCCCCTCTC
E-box(-24)-R	AGCCCCGGAGGCACCGCCCCCCGT
E-box(-74)-F	AAGGTAAACCCTCAGCCAATCAGCGGTACG
E-box(-74)-R	CCGGCCACAGCCAATCAGCAGCGC

F indicates forward primer, R, reverse primer.
