# Supplementary material for: E-Cadherin Is Transcriptionally Activated via Suppression of ZEB1 Transcriptional Repressor by Small RNA-Mediated Gene Silencing
Source: PLoS One. 2011 Dec 21;6(12):e28688. doi: 10.1371/journal.pone.0028688 (PMC3244408; doi:10.1371/journal.pone.0028688)
Supplement: Table S6 — PCR primers used in this study. (PDF) [file pone.0028688.s009.pdf]

Table S6

PCR primers used in this study.

Oligonucleotide	Sequence (5´→3´)	Oligonucleotide	Sequence (5´→3´)
E-cad promoter primer-F1	AAAGGGAGATCTTCCAGGCTAGAG GGTCACCGCGTC	E-cad promoter primer-R1	TTACACGGCGATCTTTCCGCCCTTC
E-cad promoter primer-F2	AAAGGGAGATCTGGCTGCTAGCTCA GTGGCTCATGG		
E-cad-F	CCTGGGACTCCACCTACAGA	E-cad-R	AGGAGTTGGGAAATGTGAGC
ZEB1-F	GCTGCCAATAAGCAAACGAT	ZEB1-R	CCATTTGGCTGGATCACTTT
SNAIL-F	CTATGCCGCGCTCTTTCCTC	SNAIL-R	TCGTAGGGCTGCTGGAAGGT
SLUG-F	GAGCATACAGCCCCATCACT	SLUG-R	AGGAGGTGTCAGATGGAGGA
E12/E47-F	GCCTCTCTCCAGGTCTCCTT	E12/E47-R	ACCACTGCCCTAGTTCGTGT
ACTB-F	CACACTGTGCCCATCTACGA	ACTB-R	GCCATCTCTTGCTCGAAGTC
MED8-F	CCTGCTCTCCTTTCCATCCT	MED8-R	CCCTTTCCCACATAGTCCTG
MTPN-F	TAGGTGCAGTGTGTGGAAGC	MTPN-R	TGCATGGAAGAAAACAGCAG
LATS2-F	GAACTGGTGAACGCAGGATG	LATS2-R	CTGCTTAATGACCCGCACAA
RAB31-F	CACGCTTCCACTTCACTCAA	RAB31-R	AAATGCCGCTTGCTAACAGT
GAPDH-F	TGCACCACCAACTGCTTAG	GAPDH-R	AGAGGCAGGGATGATGTTC
HNT1-F	GCTGATCTGGGCCTGAATAA	HNT1-R	ATCCCCAAAACGTGCTTAAC
PLEKHC1-F	CATGATTTGCCACAATGTCC	PLEKHC1-R	CACCCTTTTGGGCTATGTGA

F indicates forward primer, R, reverse primer.
